# Isolated pulmonary cryptococcosis in a patient with Crohn's disease treated with infliximab: A case report and literature review

**DOI:** 10.1016/j.rmcr.2021.101459

**Published:** 2021-06-24

**Authors:** Mousa Hussein, Irfan Ul Haq, Mansoor Hameed, Abbas Alabbas, Hamad Abdel Hadi, Anam Elarabi, Issam Al-Bozom

**Affiliations:** a Hamad Medical Corporation, Pulmonology Department, Qatar; b Hamad Medical Corporation, Infectious Diseases Department, Qatar; c Department of Laboratory Medicine and Pathology, Hamad Medical Corporation, Doha, Qatar

**Keywords:** Tumor necrosis factor-alpha (TNF-α) inhibitors, Infliximab, Crohn's disease, Cryptococcosis, Fungal infections

## Abstract

Tumor necrosis factor-alpha (TNF-α) inhibitors are widely used to treat various inflammatory conditions, where they have demonstrated excellent efficacy and tolerability. However, increased risk of infections is one of the most important concerns associated with these agents. Reactivation of tuberculosis and fungal infections have emerged as significant infective complications of anti-TNF-α therapy. Cryptococcus infection is an opportunistic fungal infection that can occur in patients receiving anti-TNF-α treatment. We report a rare case of isolated pulmonary cryptococcosis in a patient undergoing anti-TNF-α therapy for Crohn's disease. Our case should alert clinicians to the increased incidence and atypical presentation of pulmonary cryptococcosis in patients receiving anti-TNF-α treatment.

## Introduction

Crohn's disease (CD) is a chronic inflammatory condition affecting any part of the gastrointestinal (GI) tract. It is characterized by periods of relapse and remission. Chronic bowel inflammation often leads to complications such as fistulas and strictures. Anti-TNF-α agents are proven to help CD's most severe cases, particularly thoserefractory to steroids [1]. Anti-TNF-α agents are generally well tolerated but areassociated with various side effects, with increased susceptibility to infections being a significant concern following their initiation.

Cryptococcosis is one of the fungal infections usually diagnosed in immunocompromised patients. Long-term immunosuppressive therapy is a known risk factor for developing pulmonary cryptococcosis [[2], [3], [4]]. In Immunocompetent patients, cryptococcal infection usually results in mild chest symptoms such as low-grade fever and minimal cough, while immunocompromised patients experience more severe chest symptoms such as high-grade fever, severe chronic cough, dyspnea, and hemoptysis (5). The significant variation in the clinical and radiological manifestations of cryptococcal infection often leads to a delayed or incorrect diagnosis. A delay in the diagnosis and treatment of such infections may result in disseminated disease. We report an uncommon case of pulmonary cryptococcosis developing during therapy with infliximab in a patient with advanced Crohn's disease.

## Case report

A 54-year old male was diagnosed as having Crohn's disease in 2012 when he presented with symptoms suggestive of intestinal obstruction and underwent a right hemicolectomy and a jejunal stricture resection. Histopathology of the resected tissue showed extensive ulceration of the jejunum and the ascending colon, chronic crypt architectural distortion, and crypt abscesses. Two months later, he re-presented with abdominal pain and was found to have narrowing at the anastomotic site on coloscopy. The biopsy of the anastomotic site revealed severe inflammation and ulceration and therefore started on steroids and azathioprine.

**Fig. 1:**
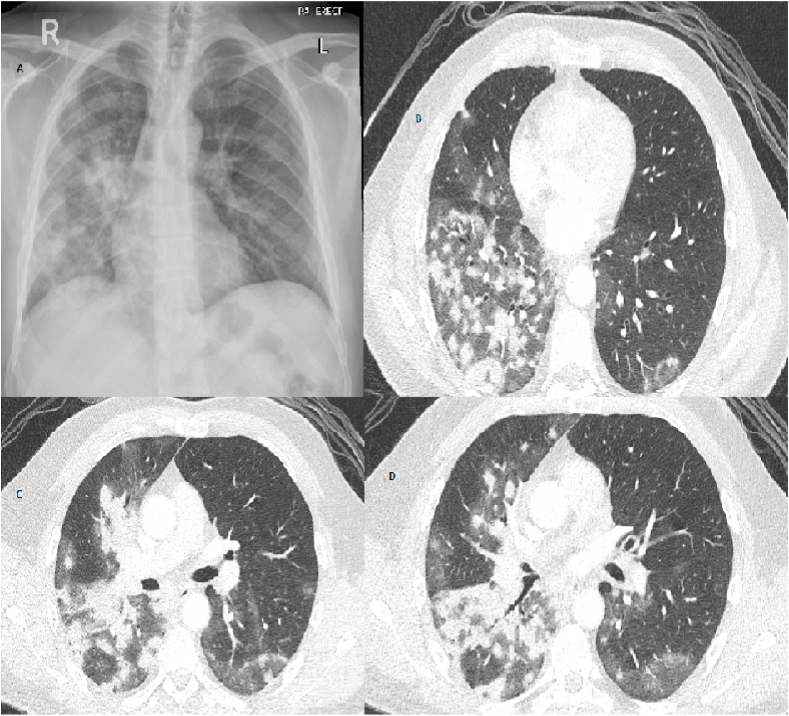
1A Chest X-ray Image showing inhomogeneous patchy infiltrates in the right mid and lower zones. (1B,C,D) CT chest Image showing multiple nodules in the right lower lobe with patchy ground-glass opacifications.

**Fig. 2:**
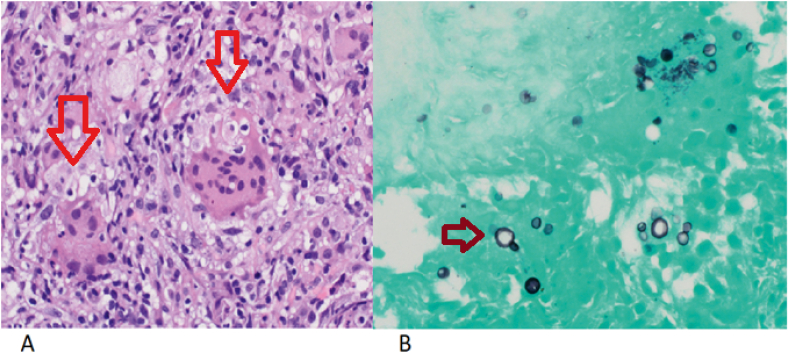
2A: lung tissue showing non-necrotizing granulomatous inflammation (arrow) 2B showing cryptococcus organism with the characteristic thick capsule as stained by GMS fungal stain.

Azathioprine was later substituted with methotrexate due to gastrointestinal intolerance and leucopenia. He was started on infliximab in July 2019 (5 mg/kg Intravenous every eight weeks) because of his progressive disease. In October 2020, he was admitted to our department with a one-month history of fever, fatigue, and productive cough with yellowish sputum. He was febrile with a temperature of 39 °C, and a chest examination revealed bilateral coarse crackles over mid-lower lung zones. He had no pets and no known contact with birds. His laboratory investigations are summarized in (Table 1). A Chest x-ray showed a right-sided ill-defined nodular infiltrate (Fig. 1A). He was initially treated as a community-acquired chest infection with ceftriaxone and azithromycin. A computerized tomography (CT) chest was requested as there was an inadequate clinical response to treatment. The CT scan revealed scattered nodules and patchy ground-glass opacities in the right lower lobe (Fig. 1B,C,D). Subsequently, bronchoscopy with transbronchial biopsies from the right lower zone was performed. Bronchoalveolar lavage showed 55% lymphocytes and a positive culture for cryptococcus neoformans. Histopathology was noticeable for non-necrotizing granulomatous inflammation, and Grocott stain identified rounded organisms with a thick capsule consistent with cryptococcus neoformans (Fig. 2). Based on the absence of neurological symptoms, lumbar puncture was not carried out. He was started on a 6- month course of daily oral fluconazole. At one month's follow-up, he reported total resolution of his symptoms with a chest X-ray showing mild regression of the right-sided infiltrates.

**Table 1:** Relevant lab investigations, including infection workup.

Investigation	Result	Normal range
WBC count	4.9	4–10 × 10^3/uL
Platelet count	290	15–400 × 10^3/uL
Hb	14	13-17 gm/dL
Eosinophil count	0.1	0.0–0.5 × 10^3/uL
Creatinine	66	62–106 μmol/L
Sodium	139	136–145 mmol/L
Alanine aminotransferase	19	0–41 U/L
C- Reactive protein	108	0–5 mg/L
Procalcitonin	0.12	<0.5 ng/ml
Albumin	30	35-50 gm/L
Lactate dehydrogenase	169	135–225 U/L
Total protein	69	66-87 gm/L
QuantiFERON gold plus	Negative	
Blood cultures	No growth	–
Urine culture	No growth	–
Common Viruses panel	Negative	–
Sputum AFB smear, PCR, and culture	Negative	–
SARS-Cov 2 PCR	Negative	–
HIV antigen/antibody ELISA	Non-reactive

## Discussion

Pulmonary cryptococcosis is an opportunistic fungal infection that has become an emerging disease in immunocompromised and immunocompetent patients. It is caused by encapsulated fungi cryptococcus gattii and C. neoformans commonly found in bird droppings, soil, and decaying wood. However, the source of the infection is not always evident, like in our case [6].

Cryptococcal infections in humans likely occur when the organism is aerosolized and inhaled. Human disease caused by this fungus ranges from asymptomatic pulmonary colonization to life-threatening meningitis and overwhelming cryptococcemia [7]. Tumor necrosis factor-alpha (TNF-α) inhibitors are increasingly being used for various inflammatory diseases. The use of these agents is associated with an increased risk of opportunistic infections like non-tuberculous mycobacteria, fungi (Pneumocystis jiroveci, Candida sp, Aspergillus, Cryptococcus, Histoplasma), opportunistic bacteria (Nocardia), parasites (Leishmania), and virus (e.g., Cytomegalovirus, Human Herpesvirus 8 [HHV 8]) infections. These infections usually occur within the first months of therapy [8]. The proposed mechanism for the increased risk of fungal infections in patients taking anti-TNF-α suppresses T-helper cells type 1 and reduces interleukin-12 and interferon-gamma [9]. Cryptococcosis was observed after a median interval of three months in many cases [10]. Our patient developed respiratory symptoms after receiving five infusions of infliximab. However, the simultaneous use of other immunosuppressive agents (steroids and azathioprine) might.

Have decreased the immune response and contributed to the development of cryptococcosis in our patient.

Pulmonary cryptococcosis usually presents with nonspecific symptoms of cough, dyspnea, chest pain, and fever. The radiological presentation can range from asymptomatic nodular disease to diffuse interstitial pattern making differentiation from other respiratory disorders quite challenging [11]. Identifying a positive culture of cryptococcus from bronchoalveolar lavage (BAL) together with the typical findings on tissue biopsies are the vital diagnostic approaches [12]. Histological staining with hematoxylin and eosin (H&E), Grocott or Gomori methenamine silver (GMS), and periodic acid-Schiff (PAS) are used to detect cryptococcus that appears as narrow-based budding yeasts (4–10 μm). Antigen tests for cryptococcus from blood or culture are occasionally positive in disseminated cryptococcal infection [12]. The role of lumbar puncture in non-HIV-infected patients with cryptococcosis is debatable but can be deferred in patients without meningeal signs [13]. We preferred not to do a lumbar puncture on our patient as he didn't have any neurological signs or symptoms.

After a careful PubMed literature review, we found only five pulmonary cryptococcosis cases in patients with Crohn's disease treated with anti-TNF-α therapy in the last 15 years. The clinical characteristics of pulmonary cryptococcosis in these patients are compared with our case (Table 2). One of the six patients received adalimumab, while the rest were treated with infliximab. It is noteworthy to mention that five out of 6 patients received other immunosuppressive therapy in addition to anti-TNF-α therapy as well. Toruner et al. in their paper, reported that infliximab, when used in combination with steroids and Azathioprine/6-mercaptopurine, is associated with an increased risk of opportunistic infections [14].

**Table 2:** Clinical characteristics of pulmonary cryptococcosis in Crohn's disease patients receiving TNF-a inhibitor.

Study Reference	Age in years	Sex	Symptoms	Anti TNF -alpha therapy	Duration of therapy/Number of doses	Concurrent Immunosuppressive therapy	CT chest findings	Method of diagnosis	Bird related exposure
Rehman et al. 2008 [15]	61	Male	None	Infliximab	2.5 Years	prednisolone, azathioprine	Multiple Nodules	Lung Biopsy	no known exposure
Osawa and Singh 2010 [16]	53	Male	Fever, diarrhea	Infliximab	Three years	prednisolone, azathioprine	Multiple Nodules	Colon Biopsy CSF culture	no known exposure
Hirai et al., 2011 [17]	39	Male	None	Infliximab	Five doses	None	Left upper lobe Nodule	Lobectomy	pigeons
Takazono et al. 2012 [18]	35	Male	High grade fever	Infliximab	Eight doses	prednisolone,	Left lower lobe Nodule	BAL cryptococcal antigen	no known exposure
J-B. Fraison et al. [19]	54	Male	Fever, cough, dyspnea	Adalimumab	Three doses	prednisolone, azathioprine	Multiple nodules, Enlarged subcarinal lymph node	Positive BAL culture	chicken manure
Present case	54	Male	Fever, cough, fatigue	Infliximab	Five doses	prednisolone, methotrexate	Right lower lobe nodules	Lung biopsy	no known exposure

The drug of choice for isolated pulmonary cryptococcosis is fluconazole [20]. Alternative agents include oral itraconazole or voriconazole [5]. Monitoring of serum cryptococcal antigen titers is not required. Pulmonary cryptococcosis in non-HIV patients has a good prognosis if properly managed. Pulmonary cryptococcosis has not led to any deaths, relapses, or dissemination among non-HIV patients in China, with a follow-up of 2–11 years [21].

## Conclusion

Isolated pulmonary cryptococcal infections, although rare, can occur in patients receiving anti-TNF-α therapy to treat underlying inflammatory conditions like Crohn's disease. Clinicians need to be aware that it can present with an atypical clinico-radiological picture, resulting in delayed diagnosis and exposing one to the possibility of disseminated infection.

## Declaration of competing interest

There is no conflict of interest for this publication by all authors.
